# Using radiation safely in cardiology: what imagers need to know

**DOI:** 10.1136/heartjnl-2017-312493

**Published:** 2019-02-18

**Authors:** Michelle Claire Williams, Christina Stewart, Nicholas W Weir, David E Newby

**Affiliations:** 1 Centre for Cardiovascular Sciences, University of Edinburgh, Edinburgh, UK; 2 Department of Medical Physics, Royal Infirmary of Edinburgh, Edinburgh, UK

**Keywords:** cardiac procedures and therapy, cardiac imaging and diagnostics, advanced cardiac imaging, cardiac catheterization and angiography, cardiac computer tomographic (ct) imaging

**Learning objectives**

- To understand how radiation dose is measured in cardiac imaging and the potential risks of radiation exposure.
- To understand the key principles of radiation protection and the concept of ‘As Low As Reasonably Practicable’.
- To understand how radiation dose can be minimised in different imaging techniques, including the general principles of patient-tailored imaging, good operator technique and improvements in hardware and software.

## Introduction

Exposure to ionising radiation is an important healthcare concern, primarily due to the potential increased lifetime risk of malignancy. This is important for patients and staff who are exposed to ionising radiation during diagnostic imaging or interventional procedures. Ionising radiation refers to radiation in the electromagnetic spectrum, which has enough energy to remove electrons from an atom. For the purposes of diagnostic imaging, this includes X-rays and gamma rays.

The UK average background radiation dose is 2.7 mSv per year, and about 0.4 mSv (16%) of this is from diagnostic medical examinations.^1 2^ In the UK, the number of CT scans performed increased fivefold between 1996/1997 and 2012/2013.^3^ Similar trends are seen in other countries and with other imaging modalities. The USA has seen a threefold increase in the annual number of nuclear medicine procedures, and CT procedures have increased 20-fold between 1985 and 2005.^4^ Cardiac imaging and interventional procedures are responsible for approximately 40% of the US cumulative effective dose due to medical imaging.^5^

### Estimating patient radiation dose

The term ‘radiation dose’ can refer to one of several measures (table 1). Dose area product (DAP) is used in X-ray and fluoroscopic imaging (eg, invasive coronary angiography [ICA]). Radiation dose in CT is calculated from dose indices measured in standardised phantoms. Volume CT dose index (CTDI_VOL_) can be used to compare protocols, and dose length product (DLP) can be used to compare doses between patients for the same CT examination. In nuclear medicine, radiopharmaceuticals emit radiation in the form of gamma rays or particles, and administered radioactivity is measured as nuclear decays per second (becquerels [Bq]).

**Table 1:** Radiation dose parameters

Modality	Parameter	Definition	Units
General	Absorbed dose.	Amount of energy deposited in a material per unit mass.	Gray (Gy) 1 Gy=1 joules per kilogram.
	Equivalent dose.	Absorbed dose multiplied by weighting factor based on the type of radiation (weighting factor of 1 for X-rays and gamma rays).	Sievert (Sv).
	Effective dose.	Whole body quantity based on absorbed organ doses weighted based on their radiation sensitivity and type of radiation; weighted sum of the organ equivalent dose.	Sieverts (Sv).
Fluoroscopy	Kerma (kinetic energy released per unit mass).	Energy transferred per unit mass of irradiated material.	Gray (Gy).
	Air kerma.	Energy transferred per unit mass of air measured with an ionisation chamber.	Gray (Gy).
	Dose area product.	Product of the air kerma and X-ray beam area.	Gy cm^2^.
	Peak skin dose	Accumulated absorbed dose to the most irradiated area of skin.	Gray (Gy).
	Fluoroscopy exposure time	Cumulative time fluoroscopy is used.	Seconds/minutes.
CT	CT dose index (CTDI).	Average absorbed dose from one axial CT scan measured with an ionisation chamber	Gray (Gy).
	Weighted CTDI (CTDI_w_).	CTDI weighted across the field of view with 1/3 for the centre and 2/3 for the edge.	Gray (Gy).
	Volume CTDI (CTDI_VOL_).	CTDI_w_ divided by pitch.*	Gray (Gy).
	Dose length product.	CTDI_VOL_ multiplied by total scan length.	mGy cm.
Radioisotopes	Radioactivity.	Rate of nuclear decay events (decays per second).	Becquerel (Bq).

*Pitch=table movement per rotation/slice thickness.

Effective dose is widely used when discussing radiation as it can be calculated for all modalities and gives an overall indication of risk from an exposure. However, it was designed for use in radiation protection within populations and only considers cancer risk. The International Commission on Radiological Protection (ICRP) has produced tissue weighting factors to reflect relative sensitivities of tissues to the carcinogenic effects of radiation (table 2). These are averages for all ages, genders and body sizes and assume that the risk is the same as if the absorbed dose was distributed uniformly throughout the body. Effective dose is calculated by multiplying the average equivalent dose in each exposed tissue by a tissue weighting factor and summing these values over the whole body. This is normally done using Monte Carlo simulation software. A more rapid method involves multiplying a displayed dose indicator (eg, DAP or DLP) by a conversion factor for a given modality and imaged anatomical region.

**Table 2:** ICRP 103 tissue weighting factors (modified from ref 49)

Tissue	Weighting factor
Gonads	0.8
Bone marrow	0.12
Colon	0.12
Lung	0.12
Stomach	0.12
Breast	0.12
Remainder tissues*	0.12
Bladder	0.04
Oesophagus	0.04
Liver	0.04
Thyroid	0.04
Bone surface	0.01
Brain	0.01
Salivary glands	0.01
Skin	0.01

*The ‘remainder tissues’ refers to the combination of the adrenals, extrathoracic region, gallbladder, heart, kidneys, oral mucosa, pancreas, prostate, small intestine, spleen, thymus, uterus, lymph nodes and muscle. All of these tissues together are assigned a weighting factor of 0.12.

ICRP, International Commission on Radiological Protection.

For CT, the most widely used conversion factor is 0.014 mSv/mGy.cm. However, this is based on out-of-date tissue weighting factors, was derived using old CT technology and was designed for chest CT, which includes a different proportion of radiosensitive tissues compared with CT coronary angiography (CTCA).^6^ Therefore, this conversion factor underestimates radiation dose. A study using computer models of patients scanned with two scanners from one manufacturer showed that 0.028 mSv/Gy.cm is a more appropriate conversion factor.^6^ This is convenient as effective dose values calculated with the previous conversion factor can simply be doubled. A recent phantom study showed that conversion factors range from 0.020 to 0.043 mSv/Gy.cm depending on CT scanner type, protocol and tube voltage.^7^ The average was 0.026 mSv/Gy.cm, and this has been proposed as a more appropriate conversion factor for CTCA.^7^

There are similar issues with the calculation of conversion factors for ICA and nuclear imaging. Ideally, the conversion factor for ICA would consider screening time at different projection angles. However, it is more convenient to use a single conversion factor based on the average for commonly used projections. Conversion factors for DAP in ICA range from 0.18 mSv /Gy.cm^2^ to 0.24 mSv/Gy.cm^2^.^8^ In nuclear imaging, the effective dose can be calculated by multiplying the activity of administered radiopharmaceutical by a tracer-specific conversion factor.^9^ Uncertainties in dose estimation in nuclear imaging include errors in measuring administered activity and differences in patient pharmacokinetics and body habitus.

The choice of conversion factor has important implications for quoted effective doses, and therefore, the factor used must always be quoted. At each step of the calculation, uncertainties can be introduced and, for an individual patient, uncertainties in effective dose may be ±40%.^10^

Typical effective doses for common procedures (table 3) vary widely, due to differences in equipment, protocols and demographics.^11^ Further standardisation, audit and quality improvement is essential to reduce radiation dose and improve consistency. In addition, it should be remembered that stated radiation doses are estimates rather than precisely known values.

**Table 3:** Estimated radiation dose of non-invasive and invasive cardiac imaging for typical equipment in typical patients^24 31 44 45 50–53^

Modality			Effective dose (mSv)
X-ray	Chest X-ray^51^		0.02
CT	Coronary artery calcium score CT^31^		1–3
	Low dose coronary artery calcium score CT*^52^		0.2–0.4
	CTCA^31^		2–5
	TAVI CT assessment (chest, abdomen and pelvis)^24^		5–50
Fluoroscopy	ICA^24^		2–20
	TAVI, transapical^24^		12–23
	TAVI, transfemoral^24^		33–100
	Diagnostic electrophysiology study^24^		0.1–3
	Radiofrequency arrhythmia ablation^24^		1–25
SPECT†‡	^99m^Tc-sestamibi 50	Stress only full dose	10
		Rest and stress half dose	6
		Rest and stress full dose	13
	^99m^Tc-tetrofosmin 50	Rest and stress half dose	6
		Rest and stress full dose	11
	^201^Thallium 44	Rest and stress half dose	10.4
		Rest and stress full dose	21
PET (rest or stress imaging)†‡	^13^N-ammonia^45^		2
	^15^O-water^45^		2
	^82^Rubidium chloride^45^		3
	^18^F-FDG^53^		5
	^18F^-Sodium fluoride^53^		4

*Using iterative reconstruction and tube voltage of 100 kV or below.

†If attenuation correction CT is performed this radiation dose must also be taken into account (~0.5–2 mSv).^45^

‡Typical values based on recommended tracer injected activity for standard patients.

18 F-FDG, ^18^F-fluorodeoxyglucose; CTCA, CT coronary angiography; ICA, invasive coronary angiography; SPECT, single-photon emission CT; TAVI, transcatheter aortic valve implantation.

### Risks of ionising radiation

Ionising radiation has the potential to cause biological harm either by directly damaging molecules, such as proteins or DNA, or through the secondary effects of free radicals generated by ionisation. Risks can be divided into stochastic (random) and deterministic (non-stochastic) effects. These can be somatic, in the individual exposed or hereditary, affecting germ cells. Inbuilt DNA repair mechanisms can mitigate radiation effects. Biochemical markers of DNA damage and repair have been identified after CTCA, ICA and single-photon emission CT (SPECT),^12–14^ but these revert back to normal background levels after 1 day.^13^

#### Deterministic effects

Deterministic effects, such as skin erythema and hair loss, have a threshold level below which they will not occur and above which the severity of the effect increases with increasing dose (table 4). It is rare for these levels to be exceeded during normal diagnostic imaging. However, for patients undergoing lengthy interventional procedures with prolonged fluoroscopic imaging in one position, the threshold for skin damage may be exceeded. Recent epidemiological studies have indicated that the threshold dose for the formation of lens opacities is lower than previously thought, at 0.5 Gy for both acute and protracted exposures. The latter is of particular importance for interventional cardiologists and electrophysiologists and is the reason why lead eye protection is recommended.

**Table 4:** Threshold levels for deterministic effects of radiation. (adapted from^54^)

Deterministic effect	Absorbed dose threshold (Gy)*
Skin erythema	3–6
Skin burns	5–10
Temporary hair loss	4
Sterility	3–6
Cataracts	0.5

For acute exposures: the time to develop these effects after exposure varies from 1 week for skin changes to 20 years for cataract development.

#### Stochastic effects

Stochastic effects, such as the development of malignancy and germ cell mutations, do not have a threshold level and may occur at any radiation dose. Their likelihood, but not severity, increases with increasing absorbed dose. The ‘linear no threshold’ (LNT) model is widely used to relate radiation dose to the risks of these effects. In the LNT model, risk increases linearly with radiation dose, and there is no threshold below which they do not occur. Evidence for the stochastic effects of radiation at doses greater than 100 mSv comes from epidemiological studies (eg, survivors of atomic weapon explosions and radiation accidents) and animal models. However, limitations with these data include the different types of radiation, variation in absorbed doses, acute and protracted exposures and the lack of evidence at radiation doses below 100 mSv. The inherent latency between a radiation exposure and the development of malignancy further complicates matters. Solid malignancies typically have a latency of 10–20 years, compared with lymphoma or leukaemia, which have a shorter latency of 2–5 years.^15^

**Figure 1:**
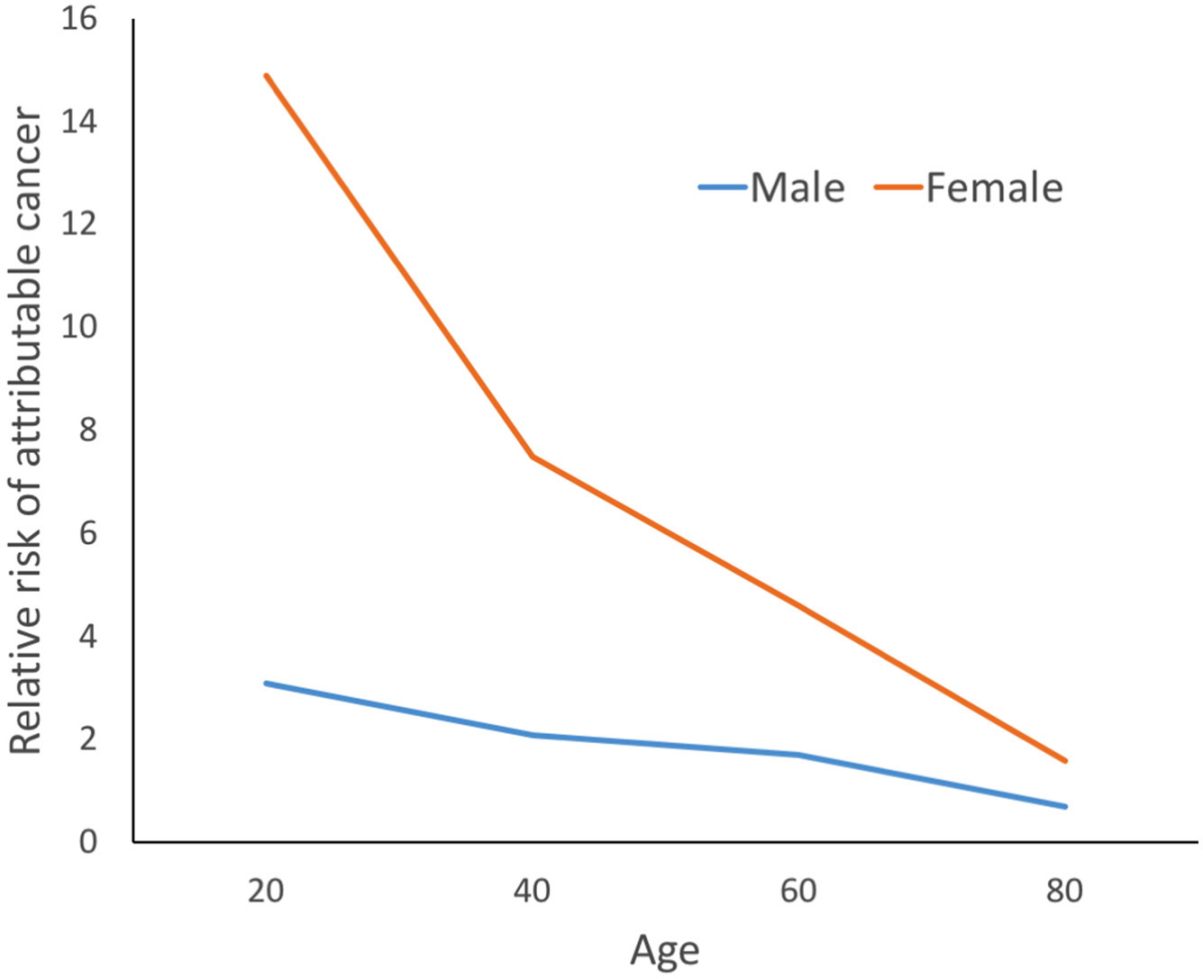
Estimated relative risk of attributable cancer from CT coronary angiography (CTCA) at different ages (compared with an 80-year-old man undergoing CTCA). Values obtained from ref 55.

The background risk of cancer is high, with a lifetime risk of cancer in the UK of approximately 50%. Therefore, to assess the effect of low-dose radiation would require very large epidemiological studies.^16^ The National Research Council Biological Effects of Ionising Radiation VII committee report has produced models that assess the potential lifetime attributable risk of cancer from medical radiation exposures.^17^ The risk of malignancy is higher for younger patients and women (figure 1). Using these models, the estimated additional lifetime risk of cancer from CTCA with a dose of 3.7 mSv would be approximately 1 in 7000 for a 50-year-old man and 1 in 2900 for a 50-year-old woman.^18^ As a rough estimation, the risk of fatal malignancy from a radiation exposure can be estimated as 5% per Sv or 1 in 20 000 per mSv.^19^ It has been estimated that 0.59% of the cancers in the UK in 2010 were due to diagnostic radiation exposures.^20^ However, this must be balanced against the health benefits of performing these tests.

### Principles of radiation protection and regulations

The central principles of radiation protection as applied to medical exposures are justification, optimisation and limitation. Justification refers to the consideration that the benefits of exposure to ionising radiation should sufficiently outweigh the risks. Appropriateness criteria and national guidelines can aid determination of the suitability of imaging or procedures for individual patients. Individual patient factors must also be considered. Optimisation refers to using the minimum amount of radiation to achieve an adequate diagnosis or procedure. This has been described as keeping radiation dose ‘As Low As Reasonably Practicable’ (ALARP). Limitation means that the effective dose to an individual should not exceed the recommended dose limits.

In the UK, legislation concerning the use of ionising radiation for medical exposures includes the Ionising Radiation Regulations 2017 and the Ionising Radiation (Medical Exposure) Regulations 2017.

#### Ionising Radiation Regulations (IRR)

The aim of the IRR is to ensure that the dose to staff and members of the public due to the use of radiation in the workplace is kept as low as reasonably practicable. This is the duty of the employer. Doses are minimised by the use of engineering controls, systems of work and personal protective equipment. IRR prescribes annual dose limits for individuals, which must not be exceeded. Following recommendations from the ICRP, the annual dose limit to the lens of the eye has been reduced from 150 mSv to 20 mSv per year in the most recent update to the regulations.

#### Ionising Radiation (Medical Exposure) Regulations (IR(ME)R)

IR(ME)R covers the justification of medical exposures involving ionising radiation and the optimisation of radiation dose. Key roles are identified with respect this: the employer, referrer, practitioner and operator. The referrer requests the investigation or procedure and must provide enough information to enable this. The practitioner assesses whether the examination is justified based on this information and, if appropriate, authorises it. The operator carries out the practical aspects of the exposure. The employer determines the entitlement to carry out these roles, within the constraints established by IR(ME)R. IR(ME)R also includes requirements for training, quality assurance of procedures and equipment, optimisation of exposures and notification requirements.

IR(ME)R established the requirement for diagnostic references levels (DRLs). DRLs are dose levels for typical examinations based on standard-sized patients, within a restricted weight range of 50–90 kg. In the UK, DRLs are calculated as the rounded third quartile value of the distribution of dosimetric or activity values for the examination being considered. National DRLs are available for a range of commonly performed procedures (table 5). DRLs are only available for some common examinations, and there is not currently a national DRL for CTCA. DRLs do not represent an ‘average’ radiation dose and are not a dose limit. Instead they can be used as an indication of good practice in order to benchmark local practices. Under normal circumstances, individual exposures are not expected to exceed DRLs consistently, but this must be considered in conjunction with other aspects of justification, including clinical factors such as patient body habitus, procedural complexity or net benefit to the patient. Examples where DRLs may be exceeded would include ICA with complex intervention for chronic total occlusion or when the local population body mass index is higher than the restricted weight range used to calculate the DRL.

**Table 5:** Diagnostic reference levels^23^

	DAP per exam (Gy cm^2^)	Fluoroscopy time per exam (min)
Coronary angiography	31	4.3
Coronary graft angiography	47	13
Percutaneous transluminal coronary angioplasty (single stent)	40	11.3
Pacemaker (permanent)	7	6

DAP, dose area product.

IR(ME)R now includes a duty for the employer to have a programme of radiation equipment quality assurance and to ensure that prior to an exposure information is provided on the benefits and risks to the individual. Additionally, the administration of radioactive substances is now governed under IR(ME)R, with applications for licences currently managed by the Department of Health and Social Care.

### Safe use of radiation in specific modalities

#### Safe use of fluoroscopy

Fluoroscopy is used in ICA, percutaneous coronary intervention, device implantation and electrophysiology. Optimisation of radiation exposure during fluoroscopy minimises the radiation dose to both patients and staff. Radiation dose can be minimised with hardware improvements, software improvements and good operator technique. Technological advances contributing to dose reduction include automated exposure control, spectral beam shaping filters, pulsed fluoroscopy, flat panel detectors, ‘last image hold’, fluoro-loops and fluoro-save features.^21^ Good operator technique includes appropriate collimation (reduction in size) of the primary beam, minimising exposure time and keeping the detector close to the patient.^22^ Viewing saved fluoro-loops rather than repeated screening reduces radiation dose. Oblique fluoroscopic angles increase the radiation dose.^23^ Real-time feedback to staff can help improve understanding of radiation dose, particularly for trainees.

Staff radiation exposure during fluoroscopy comes from the primary beam and scattered radiation. The transradial approach has a slightly increased operator radiation exposure compared with the transfemoral approach due to the higher patient radiation exposure, proximity of the operator to the X-ray tube and the less effective position of ceiling mounted shielding.^22 24 25^ Subclavian access means an even closer operator position and thus higher radiation dose.^24^ The simplest method to reduce radiation dose to staff is to use the ‘inverse squared law’, that is, doubling the distance between the operator and radiation source reduces dose by a factor of four. Further dose reduction is enabled by the use of appropriate personal protective equipment such as aprons, thyroid shields, glasses, hats, gloves and shin covers made using lead-equivalent material. At 80 kV, an 0.35 mm lead equivalent apron will transmit 3.1% of the radiation exposure. It is important that aprons are well fitting and close at the sides, as operators are often angled towards the X-ray tube. Lead aprons and eye protection should be checked annually for defects and carefully stored. Shielding to reduce staff exposure to scattered radiation includes ceiling mounted shields, lead table skirts and patient drapes. Shielding in the walls and doors of the fluoroscopy room aim to reduce the radiation dose to those outside the controlled area to well below the public dose limit. Particular care must be taken during complex interventions where prolonged screening and the presence of extra staff increases occupational exposures. Careful planning can reduce exposure times, such assessing implantation angles on CT prior to transcatheter aortic valve implantation (TAVI) or electroanatomical mapping for ablations. Dose monitoring for staff can be performed with passive or active personal dosimetry monitors. Various types of these are available to monitor whole body, collar, lens or extremity dose.

The average occupational whole body exposure for UK cardiologists in 2009/2010 was 0.12 mSv.^2^ Occupational whole body exposure for interventional cardiologists and cardiac electrophysiologists can be two to three times higher than diagnostic radiologists.^26^ However, the effect on subsequent morbidity and mortality is uncertain. A study of 43 763 radiologists and 64 990 psychiatrists showed an increase in melanoma, non-Hodgkin’s lymphoma and cerebrovascular disease among radiologists practising before 1940 but no excess mortality in those who started practising after 1940.^27^ Another study identified increased leukaemia mortality among men performing fluoroscopy guided interventions who had graduated before 1940 but no overall increase in mortality compared with psychiatrists.^28^ A study of 90 957 technologists performing fluoroscopy between 1994 and 2008 found an increase in brain cancer, breast cancer and melanoma.^29^ These effects may be due to radiation, but confounding factors may also be implicated.

#### Safe use of CT

CT of the heart can take several forms including non-contrast ECG-gated coronary artery calcium score (CACS), contrast-enhanced ECG-gated CTCA and CT imaging prior to TAVI (table 3). In the UK, the average patient dose for CTCA is 5.9 mSv (209 mGy.cm, conversion factor 0.028 mSv/mGy.cm).^30^ CACS uses standardised acquisition parameters in order to provide consistent assessment of calcium, and this has a radiation dose of 1–3 mSv.^31^ Low-dose CACS is possible but may overestimated or underestimated calcium scores.^32 33^ CT for patients undergoing TAVI involves assessment of the heart and vascular system, which includes CT of the chest, abdomen and pelvis. The radiation dose for this is higher than CTCA due to the faster heart rates and larger scan range.

The dose for CTCA depends on patient factors, such as heart rate and body mass index. The indication for the test affects radiation dose as, for example, assessment of coronary artery bypass grafts involves a larger craniocaudal scan range than assessment of native coronary arteries. Radiation dose reduction techniques for CTCA include prospective ECG-gating, reducing tube voltage and tube current, tube current modulation and minimising the scan range.^25^ Improved image reconstruction techniques, such as iterative or model-based reconstruction, can provide diagnostic quality images at a lower radiation dose.^34^ CTCA using state-of-the-art technology in patients with slow heart rates and low body mass index can achieve average radiation doses as low as 0.29 mSv.^2^

#### Safe use of nuclear imaging

Throughout the world, nuclear techniques are the most common form of cardiac imaging. In the USA, cardiac nuclear imaging accounted for 26% of overall medical radiation exposure in 2006.^26^ Radiation dose in nuclear imaging depends primarily on the choice of radiotracer.

A variety of radiotracers are available for SPECT imaging, with thallium having the highest radiation dose (table 3).^28^ Adjusting the injected radiotracer activity based on weight or body mass index can reduce radiation dose.^28^ Radiation dose can be further reduced by protocol improvements such as stress-only imaging, hardware improvements such as using newer solid state detectors (cadmium zing telluride and thallium-activated caesium iodide) and software improvements such as iterative reconstruction, resolution recovery and noise reduction.^29^ In addition, SPECT cameras with dedicated geometry and collimators optimised for cardiac imaging can further reduce radiation dose.^35^ Longer imaging time with lower injected activity could reduce radiation dose, but this must be balanced against movement artefacts.^36^ The CT used for attenuation correction can also be optimised to reduce radiation dose. Eight best practices have been established to reduce radiation dose in SPECT. (box 1)^32 33^ Worldwide, the mean radiation dose for SPECT myocardial perfusion is 10.9 mSv, with Europe having the lowest dose at 7.9 mSv.^27^ However, using stress only imaging and the latest technology means that doses as low as 0.99 mSv are possible.^27^

Box 1 **Eight best practices for SPECT myocardial perfusion imaging (adapted from ref 42).**

**Eight best practices**

-  Avoid thallium stress.
-  Avoid dual isotope.
-  Avoid too much technetium.
-  Avoid too much thallium.
-  Perform stress-only imaging.
-  Use camera based dose-reduction strategies.
-  Weight-based dosing for technetium.
-  Avoid inappropriate dosing that can lead to ‘shine through’ artefacts.

Positron emission tomography (PET) can be used to assess myocardial perfusion and conditions such as sarcoidosis, vasculitis and endocarditis. New tracers and new uses for established tracers are developing, such as the use of ^18^F-fluorodeoxyglucose and ^18^F-sodium fluoride. PET radiotracers generally have a lower radiation dose than SPECT tracers (table 3). Techniques to reduce radiation dose in PET include time-of-flight reconstructions, iterative reconstruction, three-dimensional acquisitions, weight or body mass index-adjusted radiotracer activity and the use of single attenuation correction CT images.^24 37^ Similar to SPECT, the attenuation correction CT must also be optimised to further reduce radiation dose.^38 39^

### Cumulative radiation dose

Many patients will undergo multiple imaging procedures over their lifetime. The effect of repeated exposures to low dose ionising radiation is uncertain. It has been suggested that patients should have a cumulative radiation dose record, but at present, it is unclear what to do with these records. The process of justification is based on benefit versus risk for the patient at that time point, rather than on their cumulative radiation dose.^40^ However, this sort of record can act as a reminder that all radiation exposures should be justified and optimised. When applied to the individual, cumulative dose records can have significant errors, but population-based dose registries may have benefits for quality improvement and research.^41^

### Congenital cardiac imaging

Patients with congenital heart disease are likely to undergo multiple exposures to ionising radiation during their lifetime. Therefore, the use of imaging modalities that do not use ionising radiation should be considered. Children are at an increased risk of malignancy due to their more rapidly dividing cells and longer life expectancy. The lifetime attributable risk of malignancy from ionising radiation in patients with congenital heart disease depends on age, sex, surgical complexity and subsequent life expectancy.^42^

The use of procedures involving low-dose ionising radiation in patients with congenital heart disease is increasing.^26^ Chest X-rays are the most frequent source of ionising radiation for children under 6 years with surgical procedures for congenital heart disease.^43^ However, cardiac catheterisations and CT account for between 81% and 95% of the cumulative radiation dose.^24^ The radiation dose reduction techniques discussed above should all be applied in children, and the protocol should be tailoring to their age or size.^44^

### Pregnancy

Cardiovascular diseases affect 1% of pregnant women, and during pregnancy, radiation risks apply to both the mother and the fetus. Imaging modalities that do not use ionising radiation may be preferable. However, if the benefit outweighs the risk, then an informed discussion between patient and clinician is essential, with involvement of gynaecology and medical physics as appropriate.

Radiation risks depend on the radiation dose and gestational age.^24 43–46^ For most X-ray imaging, the dose to the fetus is from scattered radiation, as the fetus can be kept out of the direct X-ray beam. However, for radiotracers with urinary excretion, the bladder is an important source of foetal exposure. Risks to the fetus are greatest in the 3rd to 8th week, so careful timing of investigations may reduce risks. Deterministic effects may occur at a threshold absorbed dose to the fetus above 150 mGy and include fetal malformation, mental retardation and spontaneous abortion.^24 44^ The estimated risk of childhood cancer from a dose of 1 mGy is about 1 in 17 000.^44^

The dose to the fetus from cardiac imaging is usually low with an estimate of <0.0001 mGy for a chest X-ray, 1 mGy for a prospectively gated CTCA and 0.074 mGy for an invasive coronary angiogram.^47^ More complex procedures and electrophysiology studies are associated with higher foetal doses, estimated as 0.0023–0.012 mGy per minute.^47^

The lactating breast has an increased risk of radiation-induced carcinogenesis. After nuclear imaging, the amount of radiotracer in breast milk will depend on the radiotracer and injected activity. For ^99m^Tc-tetrofosmin, 0.082% of the injected activity is excreted in breast milk.^48^ A recommendation to interrupt breast feeding may be given, depending on the radiotracer and administered activity, so that the dose to the infant is <1 mSv.

### Conclusion

Appropriate use of non-invasive and invasive imaging using ionising radiation is essential to minimise radiation exposure. The ALARP principle should be applied for both patient dose and occupational exposure. Methods to reduce radiation dose for all modalities include protocol optimisation and patient-tailored imaging. Personal protective equipment is important to minimise occupational exposure. Further technological improvements will continue to reduce radiation dose in all forms of imaging.

**Key messages**

- Medical imaging is an increasing source of population radiation exposure, with cardiac imaging and interventional procedures responsible for 40% of the cumulative effective dose due to medical imaging.
- The term ‘radiation dose’ is of little use as it can refer to one of several indices.
- Effective dose gives an overall indication of associated cancer risk but has limitations, in particular uncertainties in the calculation of effective dose for an individual patient may be ±40%.
- Risks from ionising radiation may be stochastic (random) or deterministic (non-stochastic).
- Radiation protection involves justification that the risks of the exposure are outweighed the benefits and that exposures must be optimised to be ‘As Low As Reasonably Practicable’.
- Radiation dose reduction techniques for all modalities include patient tailored imaging, good operator technique and hardware and software improvements.
- Personal protective equipment is important to minimise occupational radiation exposure.

**CME credits for Education in Heart**

Education in Heart articles are accredited for CME by various providers. To answer the accompanying multiple choice questions (MCQs) and obtain your credits, click on the ‘Take the Test’ link on the online version of the article. The MCQs are hosted on BMJ Learning. All users must complete a one-time registration on BMJ Learning and subsequently log in on every visit using their username and password to access modules and their CME record. Accreditation is only valid for 2 years from the date of publication. Printable CME certificates are available to users that achieve the minimum pass mark.
